# Socio-economic indicators and predisposing factors associated with traumatic dental injuries in schoolchildren at Brasília, Brazil: a cross-sectional, population-based study

**DOI:** 10.1186/1472-6831-14-91

**Published:** 2014-07-18

**Authors:** Maria de Lourdes Vieira Frujeri, José Angelo Junqueira Frujeri, Ana Cristina Barreto Bezerra, Maria Ilma de Souza Gruppioni Cortes, Edson Dias Costa

**Affiliations:** 1 Department of Dentistry, University of Brasília (UnB), AEC Setor G Norte Edifício Taguacenter Sala 116, Taguatinga, Brasília, DF, Brazil; 2 Department of Dentistry, University of Minas Gerais, Belo Horizonte, Brazil

**Keywords:** Tooth injuries, Prevalence, Demographic data

## Abstract

**Background:**

This study assessed the prevalence of traumatic dental injuries (TDI) and its association with sociodemographic and physical characteristics in the anterior permanent teeth of 12-year-old schoolchildren at the city of Brasília – DF, Brazil.

**Methods:**

A cross-sectional, population-based study was conducted on a sample of 1,389 boys and girls aged 12 years, enrolled in public and private fundamental schools at the Administrative Region (RA) of Brasília, Brazil, from October 2011 to September 2012. The demographic details were achieved by a structured questionnaire. The study recorded the type of damage, the size of incisal overjet, and whether lip coverage was inadequate. Sociodemographic data included sex, income and educational level of the parents or caretakers.

**Results:**

A total of 1118 schoolchildren were examined, yielding a response rate of 80.48%. The prevalence of TDI was 14.63% in public schools and 23.40% in private schools. The students did not differ according to sex, income and educational level of the parents or caretakers concerning the occurrence of traumas in permanent anterior teeth. Increased overjet and inadequate lip coverage were found to be important contributing factors for TDIs.

**Conclusion:**

In conclusion, this study showed an expressive prevalence of TDI in 12-year-old in schoolchildren at Brasília DF, Brazil. Sex and educational level of the parents were not associated with trauma. The increased overjet and inadequate lip coverage were significantly associated with dental trauma.

## Background

Traumatic dental injuries (TDI) have been extensively studied over the last few decades. They result in tooth fracture, displacement or loss, causing negative functional, esthetic and psychological effects to the individuals (children, adolescents and adults) [1-3]. Previous studies reported prevalence rates ranging from 6% to 27% in different populations [4-10]. In Brazil the prevalence varies widely, ranging from 10% to 58% [11-16]. The possible explanations for this variation include differences in places/environments, diagnostic criteria and examination methods [17].

Etiology and predisposing factors of traumatic injuries are well established in the literature. However, impact of socio-economic indicators remains conflicting and unclear [18,19]. The increased violence rates, number of car accidents and greater participation of children in sports activities contribute to make dental trauma an emerging public health problem. Also, the greater availability and access of leisure devices with potential risk have remarkably increased the number of cases [15]. Glendor [20] conducted a literature review on the etiology and reported risk factors for traumatic dental injuries and concluded that the number of causes of TDIs have alarmingly increased over the last decades. The author suggested that this phenomenon may be associated to the increased interest on the causes and also evidences the complex etiology of TDIs. The investigator also concluded that not only risk factors as overjet and inadequate lip coverage contribute to increase the TDIs, but also the complex interaction between the oral status of the patient, design of public parks and school playgrounds and human behavior. The question is to what extent these factors, together or separately, influence the risk of TDI.

Studies have consistently shown that male individuals have a higher chance of TDI than female individuals [8,10,17]. Socio-economic status has been associated with several oral diseases and conditions, such as dental caries, periodontal diseases, tooth loss, and oral cancer. Nevertheless, the association between TDI and socio-economic indicators remains unclear [14,21,22]. Although some researchers have reported that schoolchildren with lower socio-economic status are more likely to suffer TDI [2,13,14,17,19], others have shown an inverse correlation, with wealthier children having a higher risk of TDI [9,13]. A review paper concluded that there are few studies correlating TDI in permanent teeth with socio-economic indicators and that the majority did not find such association [22].

Among the physical factors, increased overjet and inadequate lip has been consistently associated with TDI [12,13,19,21,23]. A systematic review using meta-analysis stated that an overjet greater than 3 mm increases the chance of dental trauma. Other study considered that inadequate lip coverage is a more important risk factor for the occurrence of TDI than the increased overjet separately [24].

The purpose of this study was to assess the prevalence of TDI and its association with sociodemographic and physical characteristics in anterior permanent teeth of 12-year-old schoolchildren at the city of Brasília – DF, Brazil.

## Subjects and methods

This study was approved by the Institutional Review Board of the Health Sciences School at the University of Brasília, DF, Brazil. The Education Secretariat of the Government of Distrito Federal (GDF) authorized the study and provided the necessary information for the sample registry, which was updated on the examination date. The following data were obtained: name of all schools at Brasília, their addresses and total number of students registered in each school, at the age of 12 years.

A cross-sectional, population-based study was conducted on a sample of 1389 boys and girls aged 12 years, enrolled at public and private fundamental schools at the Administrative Region (RA) of Brasília, Brazil.

The sample size was calculated based on a sample error of 1.7%, significance level of 5%, prevalence of dental injuries of 20% and a population of 4,000 students aged 12 years, registered in public and private schools at Brasília, according to the school census of 2011.

**Figure 1:**
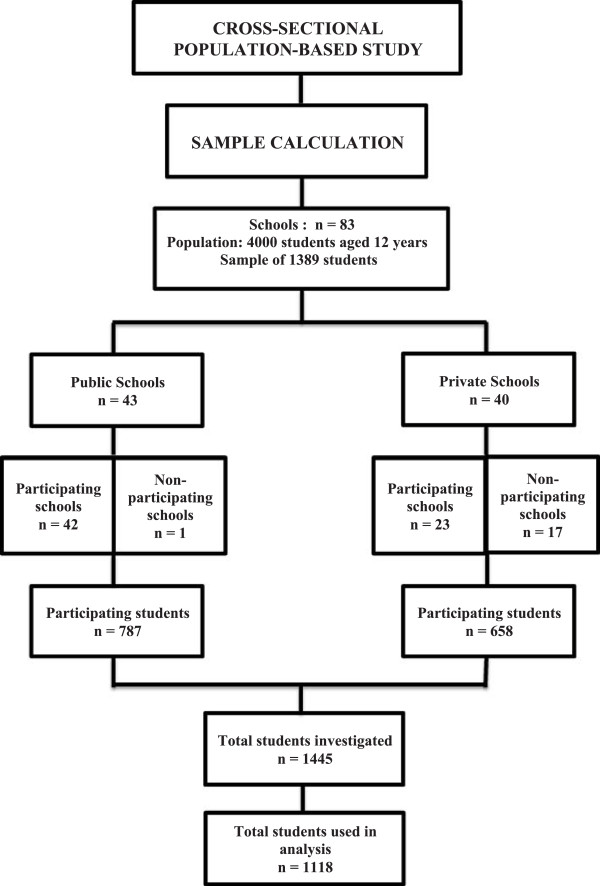
**Sample calculation and response rate of the study.** Brasília, DF, 2012.

The total of 83 fundamental schools at the administrative region of Brasília, being 43 public and 40 private, were initially contacted on the interest to participate in the study. Only one public school did not agree to participate, while only 23 private schools agreed to participate.A letter was sent to all parents or caretakers of the selected children explaining the objectives, characteristics and importance of the study. Within each school, the study was conducted only on children whose parents or caretakers signed the consent form. The final sample was composed of 787 students of public schools and 658 students of private schools, among which, in 1,118 children, it was possible to obtain information on the variables analyzed, yielding a response rate of 80.45%, based on the planned sample (Figure 1).

Socio-demographic data included the type of school (public or private), sex and educational level of the caretaker in completed years of study. The socio-economic data on the caretakers were collected by a questionnaire previously applied in another epidemiological survey [25]. This socio-economic questionnaire was used during the last Brazilian Oral Health Survey and a prevalence of 20.5% of traumatic dental injuries was found. This questionnaire was divided into four parts. The first part comprised the identification data. Similarly, in this study, we confirmed the student’s identification data, race, sex, birth date, and educational level of the parents/caretakers. The second part was composed by data on the socio-economic characteristics of the family: number of persons living in the house; number of bedrooms; material goods (TV, refrigerator, stereo, microwave oven, washing machine, number of cars; ranging from zero to ten goods); and also the family income (the sum of incomes received per each person living in the house ranging from R$ 250.00 to 9,500.00). The third part comprised questions on the educational level and years of the parents, oral morbidity and use of oral health services. And finally, the fourth part contained three questions on oral health self-perception and impacts of the parents. A section containing questions on the TDI was added to this questionnaire. TDI questions aimed to gather information on the self-perception of the parents relating to tooth traumas (first-aid notions in cases of tooth traumas, occurrence of accidents involving the mouth/teeth inside the family). If tooth trauma within the family was positively reported, we investigated which dentition was involved (primary or permanent); which type of traumatism occurred; and whether immediate care was provided. This form was sent to parents who agreed to participate in the study before clinical examination of the children “See Additional file 1.” The clinical forms and questionnaires were previously tested and did not require adjustments. A pilot study was conducted on thirty parents of the same sample to test the questionnaire. The results revealed that it was feasible in the local situation. The first thirty socio-economic questionnaires were used to test the research instrument adapted for this present study. The instrument was well understood by the participants and it was considered as effective for data collection. Thus, no adjustments were necessary. These questionnaires were included in the general sample.

Clinical data on dental trauma, lip coverage and incisal overjet were collected by oral examinations. The etiology, site of occurrence of dental trauma, age at the occurrence of trauma were obtained by direct interview with the child. The criteria for classification of trauma were the same used in the Children's Dental Health Survey at the United Kingdom [26]. These criteria include tooth fractures, discoloration and loss due to trauma to the permanent dentition. The incisal overjet was coded as smaller or equal to 5 mm or greater than 5 mm., after measurement of the greatest distance between the incisal edges of maxillary incisors in relation to the incisal edges of corresponding mandibular teeth using a CPI periodontal probe. The anterior maxillary overjet was measured with the mandibular and maxillary teeth at centric occlusion with the aid of a CPI periodontal probe placed parallel to the occlusal plane. The overjet is the greatest distance in mm between the incisal edges of maxillary incisors in relation to the incisal edges of corresponding mandibular incisors.

Anterior mandibular overjet is characterized by the anterior (labially) position of the mandibular incisors in relation to the corresponding maxillary incisors. Mandibular protrusion or crossbite was measured with the aid of a CPI periodontal probe and recorded in millimeters. Vertical open bite was characterized by lack of overlapping between maxillary and mandibular incisors.

During data collection on lip coverage, it was considered as adequate when the lips touched, entirely covering the anterior teeth, with the schoolchildren silently reading a document without knowing they were being observed. Data were collected by two dentists (Frujeri MLV and Frujeri JAJ) with help of two annotators, previously trained and calibrated at the Center of Trauma a the Federal University of Minas Gerais (UFMG). The calibration/training exercises were conducted by the professors in charge of the Center of Trauma at the aforementioned university using photographs and images of different types of traumas and patients suffering dentoalveolar traumas assisted at the clinics of this center. The degree of diagnostic reproducibility was high, the kappa coefficients for inter-examiner agreement ranged from 0.85 to 1.00, indicating almost perfect to perfect agreement, since in most cases the kappa value was equal to one.The kappa coefficients for intra-examiner agreement were all equal to 1.00, indicating perfect agreement for both examiners.

The clinical examinations were performed at the schools, during the classes, in open areas with enough natural light, with the children seated on chairs. All biosecurity procedures were strictly followed. Dental mirrors, CPI periodontal probes and gauze were packed and sterilized in sufficient numbers for one day of work. The examination included all permanent anterior teeth (incisors and canines). All teeth were dried before examination to increase the accuracy of the diagnosis. The examiner assessed existence and type of damage, treatment carried out, whether the incisal overjet was smaller or equal to 5 mm or greater than 5 mm and whether lip coverage was inadequate. The examination was conducted in a uniform fashion beginning from the maxillary right quadrant to the mandibular in clockwise direction. When the child was absent on the day of examination, a second visit was done. When tooth trauma presence was verified through clinical examination, the following characteristics were recorded into a specific sheet: type and site of injury; etiology; teeth damaged. Also, tooth trauma treatment and material type was recorded. It was recorded whether the teeth undergone trauma had not been treated until the moment of the research “See Additional file 2”. In these cases, the parents/caregivers were instructed on the importance of both trauma treatment and following-up through a letter. A pilot study was conducted on thirty schoolchildren of the same sample to test the methodology. The results revealed that it was feasible in the local situation. The inter- and intra-examiner diagnostic variability was assessed by examination in duplicate in 10% of the sample. The Kappa statistics was applied considering each tooth in each situation analyzed. These students were included in the total sample of 1,118 participants used in the analysis.

Data were entered and analyzed on the software SAS 9.2 for Windows. To evaluate if the type of overjet, lip coverage, location of the school, sex, income and educational level might explain the occurrence of trauma in permanent teeth, a mixed-effects multiple logistic regression model, with random intercept [27] was used to compensate the intra-school correlation, since the schoolchildren are clustered within schools. As a result of the model adjustment, the odds ratio and respective 95% confidence intervals were calculated.

## Results

The prevalence of dental trauma according to the variables analyzed is presented in Table 1.

**Table 1:** Prevalence of trauma to permanent teeth according to the variables analyzed in 12-year-old schoolchildren at the city of Brasília- DF- Brazil, in the year 2012

**Variables**	**Frequency (n = 1118)**	**Prevalence of trauma to permanent teeth (%)**	*CI 95%
**School**			
Public	588	14.63	11.76 – 17.49
Private	530	23.40	19.79 – 27.01
**Lip coverage**			
Adequate	989	13.65	11.51 – 15.79
Inadequate	129	58.14	49.61 – 66.67
**Overjet**			
Anterior maxilla	1064	18.89	16.53 – 21.25
Anterior mandible	11	27.27	0.91 – 53.63
Open bite	43	13.95	3.58 – 24.33
**Sex**			
Male	536	21.27	17.80 – 24.74
Female	582	16.49	13.47 – 19.51
**Educational level**			
Fundamental	210	12.86	8.32 – 17.39
High school	306	18.30	13.96 – 22.64
Graduation	494	20.44	16.88 – 24.01
Postgraduate	108	24.07	16.00 – 32.15
**Income (real)**			
Up to 250	13	15.38	0.00 – 35.03
251 – 500	67	11.94	4.16 – 19.72
501 – 1500	291	18.21	13.77 – 22.65
1501 – 2500	141	17.73	11.42 – 24.04
2501 – 4500	143	16.09	10.05 – 22.11
4501 – 9500	206	21.36	15.75 – 26.96
> 9500	257	21.40	16.38 – 26.42

*CI- Confidence Interval.

The multivariate analysis results are in Table 2.

**Table 2:** Likelihood of TDIs according to the adjusted odds ratio by mixed-effects logistic regression

**Indicators**	*OR [CI 95%]	**p-value**
**Sex**		
Male	1.33 [0.96 – 1.85]	0.08
Female	1.00	-
**Income**		0.65
Up to 250	1.00	-
251-500	1.41 [0.23 – 8.76]	0.71
501-1500	2.11 [0.39 – 11.29]	0.38
1501-2500	1.52 [0.28 – 8.39]	0.63
2501-4500	1.27 [0.23 – 7.08]	0.79
4501-9500	1.64 [0.30 – 9.10]	0.57
> 9500	1.30 [0,23 – 7.28]	0.76
**Educational level**		0.49
Fundamental	1.00	
High school	1.46 [0.86 – 2.50]	0.16
Graduation	1.37 [0.73 – 2.57]	0.33
Postgraduate	1.62 [0.73 – 3.61]	0.23
**Location**		
Private	1.53 [0.99 – 2.38]	0.05
Public	1.00	-
**Lip coverage**		
Inadequate	8.94 [5.92 – 13.51]	< 0.0001
Adequate	1.00	-
**Overjet**		0.05
Anterior maxilla	2.98 [1.15 – 7.93]	0.04
Anterior mandible	6.43 [1.02– 30.54]	0.04
Open bite	1.00	-

*OR- Odds Ratio.

The results of association studies demonstrated that students in private and public schools may have differed as to the occurrence of traumas in permanent teeth [OR = 1.53; CI 95%: 0.99-2.38; p-value = 0.05]. Concerning the gender, they did not differ regarding the occurrence of traumas in permanent teeth [OR = 1.33; CI 95%: 0.96 – 1.85]. The income and educational level did not differ concerning the occurrence of traumas in permanent teeth (p = 0.65 and p = 0.49, respectively). It was observed that students with inadequate lip coverage had 8.94 times more chances of having trauma to permanent teeth than those with adequate lip coverage [OR = 8.94; CI 95%: 5.92-13.51; p-value < 0.0001].

It was also observed that students with overjet in the anterior maxilla had 2.98 times more chances of having trauma to permanent tooth than those with open bite [OR = 2.98; CI 95%: 1.15-7.93; p-value = 0.04]. Even though the confidence interval had the value 1, it is strongly asymmetric to the right and suggests that the association for this category of overjet with trauma to permanent tooth is considerable. Thus, students with overjet in the anterior mandible had 6,43 times more chances of having trauma to permanent tooth than those with open bite [OR = 6.43; IC 95%: 1.02-30.54; p-value = 0.04].

## Discussion

The good response rate, calibration process and intra- and inter-examiner reproducibility data collaborated to the interval validity of data. The prevalence of dental trauma in the sample analyzed at Brasília was 14.63% in public schools and 23.40% in private schools. This value is relatively high if compared to other studies involving the same type of population and age. Higher values were observed at Blumenau-SC (58.6%) [19] and lower values were reported at the cities of Jaraguá do Sul –SC (15.3%) [11], Belo Horizonte-MG (13.6%) [12], Anápolis- GO (16,5%) [16], Florianópolis –SC (18.9%) [28], Campina Grande- PB (21%) [29] Recife –PE (23.3%) [13] and Herval d'Oeste –SC (17.3%) [15].

According to the literature, the male gender is at higher risk to TDI. Usually, boys are more active and perform stronger physical activities as contact sports, fights, tougher plays and use toys and equipments with higher risk potential without adequate protection. In this study the prevalence in the male gender was higher than in females, yet this difference was not statistically significant (p = 0.0850), being different from most published studies [1,29]. Some studies also did not report this difference [13,15,30]. According to a previous study, it is possible that, with the greater participation of girls in contact sports and plays, previously typical of boys, this difference might be reduced or even disappear [20].

The present results were equivocal about differences in the prevalence between children of public and private schools and also in relation to the income and educational level of the parents. Published data in the dental literature are conflicting.

Some demonstrate significant association between the prevalence and variables indicating better socio-economic condition [12,31], other corroborate the present study and did not report association [10,24], while other observed higher prevalence in children of lower socio-economic status [13,30]. There may be an interaction between the individual socio-economic condition and the physical environment. This is explained by the fact that a greater access to leisure goods and equipments may be associated to children with higher socio-economic level. For example, wealthier children have access to toys as bicycle, skate, horse riding, swimming pools and water skiing. Such equipments, when used without safety, may determine the increased prevalence. Conversely, less favored children are more exposed to public areas and recreation parks. Probably the individual mode of interaction with the environment determines the occurrence of dental trauma. Considering these inconclusive findings, further studies are necessary to elucidate the effect of the socio-economic condition on the occurrence of dental trauma.

The relationship between overjet (OJ) and TDI has been investigated by different authors [11,15,32] and demonstrate that individuals with overjet greater than 5 mm are at higher risk to TDIs compared to those with normal overjet. This study corroborates these findings, since it evidenced significant association between the presence of TDI and overjet. It was observed that students with anterior maxillary overjet greater than or equal to 5 mm had 2.98 times more chances of having trauma to permanent teeth than those with open bite, and students with mandibular anterior overjet had 6.43 times more chance of trauma to permanent teeth than those with open bite. Other studies also demonstrated this relationship [9,16]. Therefore, it may be inferred that the increased overjet is an important risk factor to dental trauma [14,33,34].

Finally, concerning the inadequate lip coverage, this study revealed similar results as other published investigations [19,35,36] that considered it as the most important and independent risk factor for the occurrence of TDIs in anterior teeth. Bonini et al. (2012) [35] observed that children with malocclusions as open bite and increased overjet, associated with inadequate lip coverage, presented high prevalence of TDIs compared to those with adequate lip coverage. It was also observed that malocclusions of anterior teeth (increased overjet and open bite) are significantly associated with TDIs only when inadequate lip coverage is present. The investigators observed that the presence of malocclusions with adequate lip coverage is not an important risk factor for TDIs. The findings of this study in the mixed-effects multiple logistic regression model corroborate the results of aforementioned investigations, since students with inadequate lip coverage had 8.94 times more chances of trauma to anterior teeth than those with adequate lip coverage (OR- CI 95% [5.92-13.51] p-value < 0.0001). We share with these investigators the idea that this possibly occurs because the lips partly absorb the impact applied to the teeth during the trauma. Since these risk factors may be corrected by orthodontic treatment, the dental professionals should clinically diagnose these risk factors and inform the children’s caretakers on the need of orthodontic intervention as early as possible [9].

## Conclusion

Sex and educational level of the parents were not associated with trauma. The increased overjet and inadequate lip coverage were significantly associated with dental trauma.

## Competing interests

The authors declare that they have no competing interests.

## Authors’ contributions

MLVF conceived of the study, participated in its design and coordination, carried out the population-based study and drafted the manuscript. JAJF participated in the design of the study, carried out the population-based study and performed the statistical analysis. ACBB participated in the design and coordination of the study as guiding the study. MISGC participated in the design of the study as co-advisor the study. EDCJ participated in the design of the study and helped to draft the manuscript. All authors read and approved the final manuscript.

## Pre-publication history

The pre-publication history for this paper can be accessed here:

http://www.biomedcentral.com/1472-6831/14/91/prepub

## Supplementary Material

Additional file 1 Socioeconomic form. Click here for file

Additional file 2 Interview and clinical examination of children participating in the survey on dental trauma. Click here for file
