# Supplementary material for: Socio-economic indicators and predisposing factors associated with traumatic dental injuries in schoolchildren at Brasília, Brazil: a cross-sectional, population-based study
Source: BMC Oral Health. 2014 Jul 18;14:91. doi: 10.1186/1472-6831-14-91 (PMC4223362; doi:10.1186/1472-6831-14-91)
Supplement: Additional file 1 — Socioeconomic form. [file 1472-6831-14-91-S1.doc]

## SOCIOECONOMIC FORM

**Research:** Maria de Lourdes Vieira Frujeri

**Advisor::** Ana Cristina Barreto Bezerra.

**Co-Orientadora:** Maria Ilma de S. G. Cortes

University of Brasília (UnB), Brazil.

College Health Sciences

department of graduate sciences

health

**Assessment of socioeconomic status, use of dental services and self-perceived oral health and dental trauma**

| **IDENTIFICATION DATA**  School:: ________________________________________________________________  Public Private  Name of student _________________________________________________________  Age: _____________ Sex: Female Ethnicity : ____________  Date: ______________ Male    Educational level of parents  Fundamental Master of Science  High school PhD  Graduation Postdoc |
| --- |

| **SOCIOECONOMIC CHARACTERIZATION OF THE FAMILY**  * indicate 9 or 99 for “don't know / did not reply”  1. How many people live in the house, including you?  2. How many rooms are used as bedrooms for the inhabitants?  3. How many goods do you have in your house? (Consider as goods: television, refrigerator, music player, microwave oven, telephone, mobile, washing machine, dishwasher, computer, and number of cars, from 0 to 11 goods)  4. In the last month, how many reais all people in your household received, including salaries, aids, retirement, rents and others? (1-Up to 250; 2-251 to 500; 3-501 to  1,500; 1,501 to 2,500; 5-2,501 to 4,500; 6-4,501 to 9,500; 7-More than 9,500) |
| --- |
| 17  **EDUCATIONAL LEVEL, REFERRED ORAL MORBIDITY AND USE OF SERVICES**  *** indicate 9 or 99 for “don't know / did not reply”**  5. Until which grade did you study? (Calculate number of years studied successfully – without fail)  6. Do you think you currently need dental treatment? (0-No; 1-Yes)  7. Did you have toothache in the last six months? (0-Yes; 1-No; 8-Not applicable)  8. A Indicate on the line beside how strong was this pain, being that (one) means little pain and 10 (ten) indicates very strong pain **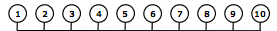**  9. Have you ever attended a dental office? (0-No; 1-Yes)  10. When did you last attend a dental office?  (1-Less than one year; 2-One to two years; 3-Three years or more; 8-Not applicable)  11. Where was your last consultation? (1-Public service; 2-Private service;  3-Health insurance; 4-Others; 8-Not applicable)  12. What was the reason for your last consultation? (1-Prevention or follow-up;  2-Pain; 3-Extraction; 4-Treatment; 5-Others; 8-Not applicable)  13. What did you think about the treatment in your last consultation?  (1-Very good; 2-Good; 3-Regular; 4-Bad; 5-Very bad; 8-Not applicable) |

| **SELF-PERCEPTION AND IMPACTS IN ORAL HEALTH**  18  * indicate 9 or 99 for “don't know / did not reply"  14. Concerning your teeth/mouth, are you: (1-Very satisfied;  2-Satisfied; 3-Nor satisfied nor dissatisfied; 4-Dissatisfied; 5-Very dissatisfied)  15.Do you think you need to use a denture or change your current denture?(0-No; 1-Yes)  16. A Some people have problems that may be caused by the teeth. Among the situations below, which are applicable for you considering the last six months?  (0-No; 1-Yes)  16.1. Did you have difficulty to eat because of toothache when drinking cold or hot beverages?    16.2. Do you feel discomfort during toothbrushing?    16.3. Do your teeth make your nervous or irritated?    16.4. Did you refrain from going out, having fun, going to parties because of  Your teeth?    16.5. Did you refrain from practicing sports because of your teeth?    16.6. Did you have difficulty to speak because of your teeth?    16.7. Did your teeth make you feel ashamed or smiling or speaking?    16.8. Did your teeth interfere with your studies or work?    16.9. Did you have poor sleep because of your teeth? |
| --- |

| SELF-PERCEPTION CONCERNING DENTAL TRAUMA  * indicate 9 or 99 for “don't know / did not reply"  17. Do you have information on first aid for dental trauma?  (0-No; 1-Yes)  18. Did someone from your family suffer accidents in the mouth and/or teeth? (0-No; 1-Yes)  18.1. If you replied yes, in which dentition:  Deciduous (milk teeth) Permanent  18.2. Which trauma occurred? ________________________________________  18.3. Was there immediate care? (0-No; 1-Yes) |
| --- |
