# Supplementary material for: Socio-economic indicators and predisposing factors associated with traumatic dental injuries in schoolchildren at Brasília, Brazil: a cross-sectional, population-based study
Source: BMC Oral Health. 2014 Jul 18;14:91. doi: 10.1186/1472-6831-14-91 (PMC4223362; doi:10.1186/1472-6831-14-91)
Supplement: Additional file 2 — Interview and clinical examination of children participating in the survey on dental trauma. [file 1472-6831-14-91-S2.docx]

University of Brasília (UnB), Brazil. Research : Maria de Lourdes Vieira Frujeri

College Health Sciences

department of graduate sciences Advisor:: Ana Cristina Barreto Bezerra.

health

INTERVIEW AND CLINICAL EXAMINATION OF CHILDREN PARTICIPATING IN THE SURVEY ON DENTAL TRAUMA

EXAMINER

| **IDENTIFICATION DATA**  School:______________________________________________ School n.__________  1- Public 2- Private  Student’s name:_________________________________________ Student n. __________  Age : _____________ SEX: 1 1- Female Ethnicity : 1- White  2- Male 2- Black  3- Asian  Address:_______________________________________________ 4-Admixture  5- Indian  Did you suffer trauma to the permanent teeth?    1- YES 2 – NO 3 – DON´T KNOW  When? ________________________________________________________________________  Where? _________________________________________________________________________  How ? _________________________________________________________________________  ________________________________________________________________________________  Date of examination:: ______________________  1- Examination performed  **2** – Examination not performed because it was not authorized by the individual or caretaker  **3** – Examination not performed, though authorized by the caretaker, because the child did not comply  **4** – Examination not performed because of absence from the school  **5** – Examination not performed for other reasons xame não realizado por outras razões |
| --- |

Trauma to the deciduous dentition (milk teeth) 1- SIM 2- NÃO 3- NÃO SABE

When? ____________________________ Where? ____________________________

How? ________________________________________________________________________

| **CAUSES (ETIOLOGY OF TRAUMA)**  **1- Fall**  a- Fall from own height  b- Fall from stairs  c- Fall in playground  d - Fall due to shove  e - Fall due to slide  f- Fall due to stumble  g - Fall from bicycle  **2- Traffic accident**  a - Car accident  b – Bicycle or motorcycle accident  c - Car crash  d- Running over by car  e- Running over by bicycle or motorcycle    **3 -**  **Accident during sports practice (cite the sport)**  **_________________________________________________________________________________**    **4 - Accident due to health problems**  a – Epilepsy  b – Cerebral palsy  c – Visual disorders  d – Hearing disorders  e – Speech disorders  f – Accident during general anesthesia (intubation)  **5 - Inadequate use of teeth**  a- Biting pencils and pens  b- Opening clasps  c- Opening plastic packages  d – Opening keys  e – Fixing some equipment  f - Opening compartments  g - Cutting or holding objects  e h– Opening bottles or cans  i - Setting up the watch  **6 - Collision against objects or people**  **7- Violence** a- Violence during playing b- Maltreatment c- Robbery  **8 - Others: _____________________________**  **9- Don´t know**    **SITE OF ACCIDENT:**  **1-**  Home 2- School 3- Street 4- Others _____________ 9 - Don´t know  Address / Site of the accident: | | | |
| --- | --- | --- | --- |
| 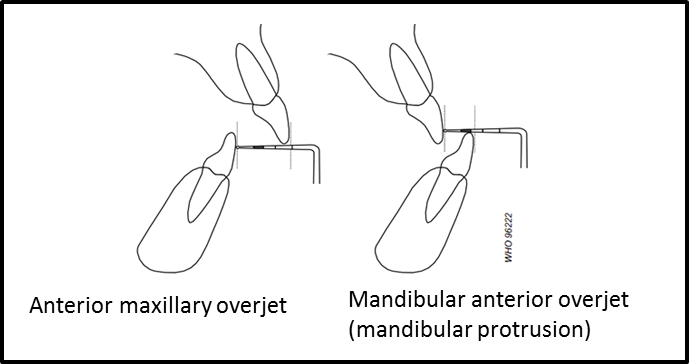  **OVERJET**  Maxillary overjet  Mandibular overjet    ≤ 5,5 mm  > 5,5 mm    Anterior vertical open bite | | |  |
| **ORAL EXAMINATION**  Previous trauma 1- Yes 2- NO    Child’s age at the occurrence of trauma:: _______________ Examination not performed     \| TOOTH \| Nº \| NUMBER OF TYPE OF LESION \| \| \| \| \| --- \| --- \| --- \| --- \| --- \| --- \| \| 13- Maxillary right canine \| 1 \|  \|  \|  \|  \| \| 12- Maxillary right lateral incisor \| 2 \|  \|  \|  \|  \| \| 11- Maxillary right central incisor \| 3 \|  \|  \|  \|  \| \| 21- Maxillary left central incisor \| 4 \|  \|  \|  \|  \| \| 22- Maxillary left lateral incisor \| 5 \|  \|  \|  \|  \| \| 23- Maxillary left canine \| 6 \|  \|  \|  \|  \| \| 33- Mandibular left canine \| 7 \|  \|  \|  \|  \| \| 32- Mandibular left lateral incisor \| 8 \|  \|  \|  \|  \| \| 31- Mandibular left central incisor \| 9 \|  \|  \|  \|  \| \| 41- Mandibular right central incisor \| 10 \|  \|  \|  \|  \| \| 42- Mandibular right lateral incisor \| 11 \|  \|  \|  \|  \| \| 43- Mandibular right canine \| 12 \|  \|  \|  \|  \| \| Others. Which?________________ \| 13 \|  \|  \|  \|  \| | | |  |
| CODE | CRITÈRIA | DESCRIPTION |  |
| 0 | No traumatic lesion |  |  |
| 1 | Discoloration |  |  |
| 2 | Enamel fracture | Fracture affecting enamel |  |
| 3 | Enamel and dentin fracture | Fracture affecting enamel and dentin |  |
| 4 | Enamel and dentin fracture with pulp exposure | Fracture affecting enamel, dentin and pulp |  |
| 5 | Missing due to trauma | Missing due to trauma |  |
| 6 | Composite restoration with acid etching | Composite restoration with acid etching, composite resin, fragment bonding |  |
| 7 | Permanent replacement | Permanent replacement with crown, removable appliance or bridge.  Specify the type:_____________ |  |
| 8 | Temporary restorations | Temporary restoration with removable appliance, with crowns or provisional bridges.  Specify the type: _____________________ |  |

Source:: Côrtes, 2000
